# Theoretische Grundlagen der klinischen Ethikberatung in der Psychiatrie

**DOI:** 10.1007/s00115-024-01730-5

**Published:** 2024-09-09

**Authors:** Jakov Gather, Matthé Scholten

**Affiliations:** 1 Klinik für Psychiatrie, Psychotherapie und Präventivmedizin, LWL-Universitätsklinikum, Ruhr-Universität Bochum, Alexandrinenstr. 1–3, 44791 Bochum, Deutschland; 2 Institut für Medizinische Ethik und Geschichte der Medizin, Ruhr-Universität Bochum, Bochum, Deutschland

**Keywords:** Klinische Ethik, Klinisches Ethikkomitee, Moralischer Stress, Philosophie, Medizinethik, Clinical ethics, Clinical ethics committee, Moral distress, Philosophy, Medical ethics

## Abstract

**Hintergrund:**

Im Rahmen klinischer Ethikberatung unterstützen Ethikberater*innen im Gesundheitswesen Professionelle in der Psychiatrie dabei, moralische Probleme zu identifizieren und zu analysieren.

**Ziel der Arbeit:**

Darstellung von zentralen ethischen Grundbegriffen und Begründungsansätzen, die für die klinische Ethikberatung in der Psychiatrie von Relevanz sind.

**Material und Methoden:**

Konzeptionelle und ethische Analyse.

**Ergebnisse:**

Nach einer Unterscheidung von Moral, Ethik und Recht werden moralische von anderen Problemen abgegrenzt. Im Anschluss werden ethische Grundbegriffe geklärt und das Konzept des moralischen Stresses vorgestellt. Im Hinblick auf ethische Begründungsansätze werden philosophische ethische Theorien von medizinethischen Theorien, wie der Prinzipienethik und der Care-Ethik, abgegrenzt. Abschließend werden Rechtfertigungstests auf Grundlage des Schadensprinzips und des schwachen Paternalismus zur ethischen Entscheidungsfindung in Situationen von Eigen- oder Fremdgefährdung erläutert.

**Diskussion:**

Die Kenntnis ethischer Grundbegriffe und Begründungsansätze ist wichtig für die Identifikation und Analyse moralischer Probleme in der Psychiatrie und sollte in der Ausbildung von Ethikberater*innen im Gesundheitswesen stärker vermittelt werden.

## Abstract (en)

**Background:**

Clinical ethics consultants support mental health professionals in identifying and analyzing moral problems in clinical practice.

**Objective:**

Presentation of key ethical concepts and normative theories that are relevant for clinical ethics consultation in mental healthcare.

**Material and methods:**

Conceptual and ethical analyses.

**Results:**

After distinguishing between morality, ethics and law, moral problems are differentiated from other types of problems encountered in clinical practice. Subsequently, key ethical concepts and the concept of moral distress are clarified. In relation to the normative framework a distinction is made between philosophical ethical theories and medical ethical theories, such as principlism and the ethics of care. Finally, justification tests for ethical decision-making in situations of danger to self or others based on the harm principle and soft paternalism are proposed.

**Conclusion:**

Knowledge of key ethical concepts and normative theories is important for the identification and analysis of moral problems in mental healthcare and should be given greater weight in the training of clinical ethics consultants.

## Hintergrund

Ethikberatung im Gesundheitswesen umfasst vielfältige Strukturen, durch die Professionelle in medizinischen Einrichtungen für ethische Fragestellungen sensibilisiert und bei der Lösung ethischer Herausforderungen in der Versorgung von Patient*innen unterstützt werden sollen [28, 33]. Letzteres erfolgt u. a. im Rahmen ethischer Fallbesprechungen, bei denen ein von Ethikberater*innen im Gesundheitswesen moderierter strukturierter kommunikativer Prozess in Gang gesetzt wird [32].

In psychiatrischen Krankenhäusern gewinnt klinische Ethikberatung zunehmend an Bedeutung [7, 35]. Ethikberater*innen im Gesundheitswesen sollen gemäß dem „Curriculum Ethikberatung im Gesundheitswesen“ der medizinethischen Fachgesellschaft Akademie für Ethik in der Medizin (AEM) ethische Grundbegriffe und Begründungsansätze kennen und in der Lage sein, moralische Probleme zu identifizieren und zu analysieren [2]. Dies setzt Kenntnisse und Fähigkeiten voraus, die über das medizinisch-psychiatrische Fachgebiet hinausreichen und erfordert Rückgriffe auf die Medizinethik und die Philosophie. Im Folgenden werden zentrale ethische Grundbegriffe und Begründungsansätze erläutert und im Kontext der Psychiatrie veranschaulicht und nutzbar gemacht.

## Was unterscheidet Moral, Ethik und Recht?

In der deutschen medizinethischen Literatur wird üblicherweise zwischen den Begriffen „Moral“ und „Ethik“ unterschieden [17]. Obwohl sich diese begriffliche Trennung nicht unbedingt im alltäglichen Gebrauch der Wörter widerspiegelt und daher für manche artifiziell wirken mag, ist sie aus theoretischer Sicht hilfreich. Folgt man dieser begrifflichen Trennung, bezeichnet „Moral“ die von Individuen oder Gruppen akzeptierten Überzeugungen und Regeln, „Ethik“ hingegen die systematische Analyse von Moral unter Verwendung wissenschaftlicher Methoden [17]. Bezogen auf die klinische Ethikberatung kann diese Unterscheidung wie folgt verdeutlicht werden: In einer ethischen Fallbesprechung werden von den beteiligten Akteur*innen bisweilen unterschiedliche moralische Ansichten zu einem konkreten Fall vertreten. Diese unterschiedlichen moralischen Ansichten werden im Rahmen der ethischen Fallbesprechung aber nicht unreflektiert im Raum stehen gelassen, sondern es wird geprüft, inwiefern die jeweiligen Ansichten begründet werden können und somit einer ethischen Analyse standhalten.

In der Philosophie wird oft zwischen zwei Hauptarten der ethischen Analyse unterschieden: Die erste befasst sich mit evaluativen Fragen des guten Lebens und die zweite mit normativen Fragen des moralisch Richtigen [17, 25]. Die erste Art der ethischen Analyse spielt in der klinischen Ethikberatung nur eine marginale Rolle. In unserer liberalen und pluralistischen Gesellschaft darf jede Person für sich selbst entscheiden, worin für sie das gute oder gelungene Leben besteht, solange sie dabei nicht den berechtigten Interessen anderer Personen schadet [20, 23]. In der ethischen Fallbesprechung kann es bisweilen erforderlich werden, die beteiligten Akteur*innen davon abzuhalten, die eigene Auffassung des guten Lebens auf die betroffene Person zu projizieren und daraus abzuleiten, wie in einer gegebenen Situation gehandelt werden müsste, um dieser zu einem guten Leben zu verhelfen.

Die zweite Hauptart der ethischen Analyse spielt dagegen eine zentrale Rolle in der klinischen Ethikberatung. So geht es bei ethischen Fallbesprechungen in der Psychiatrie z. B. häufig um die Frage, ob eine bestimmte Behandlung gegen den Willen einer Person durchgeführt werden darf. Wenn bestimmte Beteiligte der Meinung sind, eine Zwangsbehandlung durchzuführen sei in der vorliegenden Situation moralisch angemessen, andere Beteiligte dies aber bestreiten, geht es gerade darum, herauszufinden, welche von diesen beiden Ansichten ethisch zu rechtfertigen ist.

Abzugrenzen sind Moral und Ethik vom Recht, das die Gesamtheit aller staatlich festgelegten bzw. anerkannten Gesetze und gesetzesähnlichen Normen umfasst [6]. Anders als moralische Normen können Rechtsnormen auch zwangsweise durchgesetzt und Verstöße dagegen bestraft bzw. geahndet werden [15]. Das Verhältnis von Ethik und Recht erfordert gerade in der Psychiatrie eine genauere Betrachtung, da dort viele Bereiche rechtlich im Detail geregelt sind und rechtliche Entwicklungen unmittelbaren Einfluss auf die klinische Praxis haben [29]. Vor diesem Hintergrund könnte man auf die Idee kommen, dass klinische Ethikberatung in der Psychiatrie überflüssig sei, weil der rechtliche Rahmen ohnehin vorgebe, was in einer Situation erlaubt ist und was nicht. Professionelle in der Psychiatrie könnten dazu verleitet werden, moralisch schwierige Entscheidungen Gerichten überlassen zu wollen („Das soll der Richter entscheiden!“), um sich selbst moralisch nicht positionieren zu müssen. Eine solche Haltung ist jedoch unangemessen, weil der rechtliche Rahmen in der klinischen Praxis einen relativ großen Handlungsspielraum lässt, in dem Professionelle begründete Entscheidungen treffen und diese gegenüber den davon betroffenen Patient*innen moralisch verantworten müssen.

## Abgrenzung moralischer Probleme von anderen Problemen

Es ist wichtig, sich zu vergegenwärtigen, dass Professionelle in der Psychiatrie nicht nur mit moralischen, sondern mit einer Vielzahl an weiteren Problemen konfrontiert sein können. Aus diesem Grund stellen Angebote der klinischen Ethikberatung nicht für jedes Problem eine geeignete Intervention dar. Moralische Probleme müssen von anderen Problemen abgegrenzt werden, um für die jeweilige konkrete Problemlage die angemessene Problemlösestrategie auswählen zu können. Folgende Formate kommen hierbei unter anderem in Betracht:

- Psychiatrische Fallbesprechung: Bestehen Unsicherheiten bei der Beurteilung von Diagnose, Therapie oder Prognose, bietet sich eine psychiatrische Fallbesprechung an, bei der ein Behandlungsteam – idealerweise unter Beteiligung einer (ggf. externen) Person mit viel Erfahrung und Expertise im Hinblick auf die zugrunde liegende Fragestellung – einen Fall aus psychiatrisch-psychotherapeutischer Sicht systematisch beleuchtet und diskutiert.
- Rechtliche Klärung: Bestehen Unsicherheiten bezogen auf eine konkrete rechtliche Fragestellung, ist die Rücksprache mit einem Juristen oder einer Rechtsabteilung zu empfehlen.
- Supervision: Zeigen sich in einem konkreten Fall innerhalb eines Teams Schwierigkeiten in der multiprofessionellen Zusammenarbeit und resultieren daraus ggf. psychische Belastungen bei den Teammitgliedern, kann das Einbringen dieses Falls in eine Supervision sinnvoll sein [21].

Die Herausforderung in der klinischen Praxis besteht darin, dass die oben skizzierten Probleme häufig zusammen auftreten. Ein Beispiel hierfür könnte ein Patient mit einer therapieresistenten Psychose aus dem schizophrenen Formenkreis sein, der wiederholt psychotisch motiviertes fremdaggressives Verhalten gegenüber Mitarbeitenden aus der Pflege zeigt und bei dem im Team immer wieder kontrovers über die Art und Dauer von Zwangsmaßnahmen diskutiert wird. In solch vielschichtigen Fallkonstellationen empfiehlt es sich, vor Beginn einer ethischen Fallbesprechung andere Fragen – z. B. rechtliche Unklarheiten oder psychiatrische Fragen im Hinblick auf verbleibende Behandlungsoptionen – bereits weitestgehend geklärt zu haben, um sich in der ethischen Fallbesprechung auf die ethische Analyse der unterschiedlichen Handlungsoptionen fokussieren zu können.

## Ethische Grundbegriffe

In der klinischen Praxis ist oft von moralischen Problemen, Konflikten oder Dilemmata die Rede. Obwohl die genannten Begriffe häufig synonym verwendet werden, sind sie streng genommen nicht gleichbedeutend [25]. Der Begriff moralisches *Problem *hat die weiteste Bedeutung und bezeichnet eine Situation, in der es unklar ist, welche Handlungsweise moralisch angemessen ist. Der Begriff moralischer* Konflikt *stammt primär aus dem Kontext zwischenmenschlicher Konflikte und wurde danach auf den Konflikt zwischen zwei oder mehreren moralischen Normen übertragen [25]. Ein typischer moralischer Konflikt in der Psychiatrie tritt dann auf, wenn ein*e Patient*in eine medizinisch indizierte Behandlung (z. B. mit einem Antipsychotikum) ablehnt und dadurch ihr gesundheitliches Wohl beeinträchtigt wird. In einer solchen Situation kommt es zu einem Konflikt zwischen dem Prinzip des Respekts der Selbstbestimmung (welches das Befolgen der Entscheidung der Person fordert) und dem Prinzip der Fürsorge (welches die Förderung des gesundheitlichen Wohls der Person fordert). Ein solcher Konflikt kann in einem moralischen Dilemma münden. Ein moralisches *Dilemma* beschreibt eine Situation, in der es keine Handlungsoption gibt, die allen infrage kommenden moralischen Anforderungen gleichzeitig gerecht wird. Bezogen auf das gerade genannte Beispiel bedeutet dies, dass Professionelle in der Psychiatrie nicht die Selbstbestimmung der betroffenen Person achten können, ohne dass durch die ausbleibende Behandlung gleichzeitig deren Wohl gefährdet wird; und umgekehrt nicht durch Durchführung einer Behandlung zum Wohl der betroffenen Person handeln können, ohne gleichzeitig deren Selbstbestimmung zu verletzen. In einem strengen philosophischen Sinne bezeichnet ein moralisches Dilemma eine Situation, in der *keine* der zur Verfügung stehenden Handlungsoptionen moralisch gerechtfertigt wäre. Obwohl es sich kaum bestreiten lässt, dass es in der klinischen Praxis Situationen gibt, in denen Professionelle im Gesundheitswesen nicht allen relevanten moralischen Anforderungen gleichzeitig gerecht werden können, wird in der philosophischen Ethik kontrovers diskutiert, ob es überhaupt so etwas wie ein moralisches Dilemma im strengen Sinne geben kann [19].

Ziel einer ethischen Fallbesprechung in der Psychiatrie könnte sein, Professionelle in einer moralischen Dilemmasituation dabei zu unterstützen, begründete Entscheidungen für oder gegen die verschiedenen Handlungsoptionen zu treffen und eine ethisch gerechtfertigte Handlungsoption zu identifizieren. Bezogen auf das genannte Beispiel wären relevante Fragen in diesem Zusammenhang, ob die Person einwilligungsfähig ist im Hinblick auf die Ablehnung der Behandlung mit dem Antipsychotikum und ob die weiter unten genannten engen Voraussetzungen für die Durchführung einer Behandlung unter Zwang erfüllt sind.

## Moralischer Stress

Moralische Probleme, Konflikte und Dilemmata können bei Professionellen im Gesundheitswesen psychische Reaktionen hervorrufen, die unter dem Begriff „moralischer Stress“ („moral distress“) zusammengefasst werden [13]. Empirische Studien zeigen, dass moralischer Stress unter Professionellen im Gesundheitswesen weit verbreitet ist [10] und auch in der Psychiatrie eine Rolle spielt [14]. Die Entwicklung des Begriffs geht auf Andrew Jameton [12] zurück, der die Ursache des moralischen Stresses darin sah, dass eine Person durch institutionelle Umstände oder Regeln genötigt wird, entgegen ihrem eigenen Urteil darüber, was moralisch angemessen ist, zu handeln. Mittlerweile wird der Begriff „moralischer Stress“ jedoch in einem breiteren Sinne verwendet und schließt auch psychische Reaktionen ein, die aus moralischer Unsicherheit oder der Konfrontation mit moralischen Dilemmata resultieren [22]. Empirische Studien belegen, dass moralischer Stress u. a. zu Frustration, Schuldgefühlen und Erschöpfung unter Professionellen im Gesundheitswesen führen kann [22].

Die Reduktion moralischen Stresses wird als ein Outcomeparameter ethischer Fallbesprechungen diskutiert [4, 26]. Ethikberatung zielt darauf ab, moralische Unsicherheit aufzulösen und die beteiligten Akteur*innen in dem Gefühl zu stärken, in einer konkreten Situation eine ethisch gerechtfertigte Lösung für ein moralisches Problem gefunden zu haben – auch wenn letzteres nicht bedeutet, dass die involvierten Personen nicht auch bedauern können, so handeln zu müssen, wie sie müssen, oder sich nicht auch wünschen können, dass es das vorliegende moralische Problem überhaupt nicht gäbe. Daraus ergibt sich, dass auch trotz einer erfolgten ethischen Fallbesprechung unangenehme und belastende psychische Reaktionen bestehen bleiben können. Unter Umständen können letztere durch die Ethikberatung sogar verstärkt werden, etwa wenn die Beteiligten im Rahmen einer ethischen Fallbesprechung realisieren, dass sie aufgrund von Kontextfaktoren (z. B. institutionellen oder rechtlichen Rahmenbedingungen) die aus ihrer Sicht ethisch angemessenste Lösung nicht umsetzen können. Professionelle im Gesundheitswesen sollten allerdings im Anschluss an eine ethische Fallbesprechung darauf vertrauen können, in einer gegebenen und moralisch herausfordernden Situation eine ethisch gut begründete Entscheidung getroffen zu haben.

## Philosophische ethische Theorien

Nach der Identifikation eines moralischen Problems in der klinischen Praxis stellt sich die Frage, wie im Rahmen einer ethischen Analyse beurteilt werden soll, welche Handlungsweise moralisch gerechtfertigt ist. In der Philosophie und in der Medizinethik sind verschiedene ethische Begründungsansätze entwickelt worden, die bestimmen sollen, wann eine Handlung moralisch gerechtfertigt ist. Die Hauptarten philosophischer ethischer Theorien sind Deontologie, Konsequentialismus und Tugendethik. Alle drei ethischen Theorien streben eine Letztbegründung der Moral an und vertreten unterschiedliche Auffassungen im Hinblick auf moralische Richtigkeit.

Deontologischen Theorien zufolge ist eine Handlung genau dann moralisch gerechtfertigt, wenn das Prinzip der Handlung bestimmte Kriterien erfüllt. Nach der Theorie Immanuel Kants muss das Prinzip der Handlung mit dem sogenannten „kategorischen Imperativ“ übereinstimmen, der fordert, dass das Prinzip, nach dem man handelt, verallgemeinert werden kann – was bedeutet, dass die Handlung immer noch möglich wäre und zu ihrem Ziel führen würde, wenn alle Menschen nach dem Prinzip handeln würden. Pflichten für Professionelle im Gesundheitswesen, die hieraus folgen, sind u. a. die Pflichten zur Wahrhaftigkeit und zum Respekt der Selbstbestimmung.

Konsequentialistische Theorien beurteilen die moralische Qualität einer Handlung an deren Folgen. Utilitarismus ist eine Form des Konsequentialismus, dem zufolge der Nutzen der Handlungsfolgen der für die ethische Analyse relevante Parameter ist. Eine Handlung ist also genau dann moralisch gerechtfertigt, wenn die Handlung von den verfügbaren Handlungsoptionen die höchste Nettosumme des Nutzens für alle Beteiligten hat. Für Professionelle im Gesundheitswesen folgt daraus die Pflicht, Patient*innen die Behandlungsoption mit dem besten Nutzen-Schaden-Verhältnis anzubieten – und keine Behandlung durchzuführen, wenn die Option, keine Behandlung durchzuführen, das beste Nutzen-Schaden-Verhältnis aufweist.

Tugendethische Theorien bewerten eine Handlung genau dann als moralisch gerechtfertigt, wenn eine tugendhafte Person sie in der vorliegenden Situation ergreifen würde. Bei tugendethischen Theorien geht es in der ethischen Analyse also nicht primär um die Handlung oder ihre Folgen, sondern um den Charakter der handelnden Person und ihre Sensibilität für den Entscheidungskontext und die Gegebenheiten der vorliegenden Situation. Klinisch relevante Beispiele für Tugenden sind technische Fertigkeit (d. h. professionelle Kompetenz) und praktische Weisheit (d. h. die Fähigkeit, situationsgerecht entscheiden zu können).

Die klassische angewandte Ethik hat diese Theorien gewissermaßen direkt auf konkrete moralische Probleme aus der klinischen Praxis angewendet. Dieses Vorgehen hat zwei grundsätzliche Probleme: Erstens zeigt es eine gewisse Naivität, indem es voraussetzt, dass die Philosophie zu Lösungen moralischer Probleme in der klinischen Praxis kommen kann, ohne sich grundsätzlich mit dem Erfahrungswissen der beteiligten Akteur*innen auseinanderzusetzen. Zweitens hat die Anwendung philosophischer ethischer Theorien auf moralische Probleme aus der klinischen Praxis immer wieder zu einem Theorienstreit geführt. Deontologen, Konsequentialisten und Tugendethiker kommen nämlich in vielen Anwendungsfällen zu unterschiedlichen Ergebnissen und können dies leicht zum Anlass nehmen, die Vor- und Nachteile bestimmter philosophischer Theorien zu diskutieren, anstatt eine konsensfähige Lösung im konkreten Fall zu erarbeiten.

## Medizinethische Theorien

Um die erwähnten Schwächen philosophischer ethischer Theorien zu überwinden und Professionelle im Gesundheitswesen besser zur ethischen Entscheidungsfindung zu befähigen, wurden verschiedene medizinethische Theorien entwickelt. Die bekanntesten Beispiele sind die *Prinzipienethik *von Tom L. Beauchamp und James F. Childress sowie die *Care-Ethik*, die aufbauend auf Arbeiten von Carol Gilligan entstanden ist.

Die Prinzipienethik gibt den Versuch einer Letztbegründung der Moral bewusst auf und stellt vier moralische Prinzipien mittlerer Reichweite auf, die mit den unterschiedlichen philosophischen ethischen Theorien vereinbar sind und Professionellen im Gesundheitswesen dabei helfen können, moralische Probleme zu strukturieren und Lösungen zu erarbeiten. Die vier Prinzipien lauten Respekt der Selbstbestimmung („respect for autonomy“), Nichtschaden („nonmaleficence“), Fürsorge („beneficence“) und Gerechtigkeit („justice“) und können wie folgt konkretisiert werden [3]:

- Das Prinzip des Respekts der Selbstbestimmung verlangt von Professionellen im Gesundheitswesen, dass sie die Entscheidungen von Patient*innen respektieren sowie sicherstellen, dass deren Entscheidungen informiert und freiwillig sind.
- Das Prinzip des Nichtschadens verlangt von Professionellen im Gesundheitswesen, dass sie die Risiken von Schäden für Patient*innen minimieren und Schäden soweit wie möglich verhindern.
- Das Prinzip der Fürsorge verlangt von Professionellen im Gesundheitswesen, dass sie durch medizinische Maßnahmen das gesundheitliche Wohlergehen ihrer Patient*innen fördern.
- Das Prinzip der Gerechtigkeit verlangt von Professionellen im Gesundheitswesen, dass sie Patient*innen fair behandeln und die verfügbaren Ressourcen gerecht verteilen.

Beauchamp und Childress [3] unterstreichen, dass diese vier Prinzipien gleichberechtigt sind (d. h. keines der vier Prinzipien Vorrang vor den anderen hat) und dass sie im konkreten klinischen Einzelfall gegeneinander abgewogen werden müssen.

In der Care-Ethik wird der Begriff *Care* viel breiter gefasst als nur auf die medizinische Versorgung bezogen, und der Anwendungsbereich von Care-Ethik-Theorien geht demgemäß über den Anwendungsbereich der Medizin hinaus. Im Folgenden soll auf die Implikationen des Care-Ethik-Ansatzes für die Medizinethik fokussiert werden. *Care* besteht nach Tronto [30, 31] aus den folgenden vier Elementen: Achtsamkeit („attentiveness“), Verantwortung („responsibility“), Kompetenz („competence“) und Resonanz („responsiveness“).

- Das Element der Achtsamkeit kommt dann zum Ausdruck, wenn Professionelle im Gesundheitswesen auf die Bedürfnisse und Anliegen von Patient*innen eingehen, ihnen aktiv zuhören, sich in sie einfühlen und angemessen ihre kognitiven und emotionalen Ressourcen und Defizite berücksichtigen.
- Das Element der Verantwortung kommt dann zum Ausdruck, wenn Professionelle im Gesundheitswesen Patient*innen angemessen versorgen und unterstützen sowie für eine gerechte medizinische und pflegerische Versorgung einstehen.
- Das Element der Kompetenz kommt dann zum Ausdruck, wenn Professionelle im Gesundheitswesen über die erforderlichen fachlichen Fähigkeiten und Kenntnisse verfügen und sich bemühen, sich in dieser Hinsicht zu verbessern und ihre Fähigkeiten und Kenntnisse an die individuellen Bedürfnisse von Patient*innen anzupassen.
- Das Element der Resonanz kommt dann zum Ausdruck, wenn Professionelle im Gesundheitswesen auf die besonderen Bedürfnisse und Umstände individueller Patient*innen eingehen und fähig und bereit dazu sind, ihr eigenes Handeln aufgrund von Rückmeldungen von anderen oder veränderten Umständen anzupassen [30, 31].

Im Ansatz der Prinzipienethik spiegeln sich indirekt die Deontologie (vor allem im Prinzip der Selbstbestimmung) und der Utilitarismus (vor allem in den Prinzipien des Nichtschadens und der Fürsorge) wider. Im Care-Ethik-Ansatz spiegelt sich die Tugendethik wider. Eine Gemeinsamkeit der beiden medizinethischen Theorien liegt darin, dass sie bewusst keinen Entscheidungsalgorithmus zur Verfügung stellen, sondern vielmehr darauf abzielen, Professionelle im Gesundheitswesen bei der eigenen ethischen Reflexion zu unterstützen. Auf Grundlage beider Theorien sind Ansätze für die Ethikberatung entwickelt worden: Das Modell der prinzipienorientierten Falldiskussion [18] rekurriert auf die Prinzipienethik, während der Ansatz der „moral case deliberation“ theoretische Bezüge zur Care-Ethik aufweist [34].

## Situationen von Eigen- oder Fremdgefährdung

Eine besondere Herausforderung, mit der Professionelle und Ethikberater*innen in der Psychiatrie regelmäßig konfrontiert sind, sind Situationen von Eigen- oder Fremdgefährdung und damit einhergehende Fragen nach der ethischen Rechtfertigung von Zwang [1, 16, 24]. Zur Analyse derartiger Situationen schlagen wir zwei spezifische ethische Rechtfertigungstests vor, die dabei helfen können, auf strukturierte Weise ethisch begründete Entscheidungen im Zusammenhang mit Zwang zu treffen. Der Rechtfertigungstest zur ethischen Entscheidungsfindung in Situationen von Fremdgefährdung basiert auf dem sogenannten *Schadensprinzip* und der Rechtfertigungstest zur ethischen Entscheidungsfindung in Situationen von Eigengefährdung auf dem sogenannten *schwachen Paternalismus* [11]. Beiden Rechtfertigungstests ist gemeinsam, dass eine Zwangsmaßnahme nur dann moralisch gerechtfertigt ist, wenn sie geeignet, notwendig und verhältnismäßig ist, um die Gefährdung abzuwenden [5, 8, 9, 11, 27]. Demnach muss sowohl in Situationen von Eigen- als auch von Fremdgefährdung beurteilt werden,

- ob die Zwangsmaßnahme ein wirksames Mittel darstellt, um die Gefährdung abzuwenden (Kriterium der Geeignetheit),
- ob die Zwangsmaßnahme notwendig ist, d. h. es keine milderen Mittel im Vergleich zur Zwangsmaßnahme gibt (Kriterium der Notwendigkeit) und
- ob das Nutzen-Risiko-Verhältnis der Durchführung der Zwangsmaßnahme das Nutzen-Risiko-Verhältnis der Nichtdurchführung der Zwangsmaßnahme deutlich überwiegt (Kriterium der Verhältnismäßigkeit).

Jedes Kriterium ist für sich genommen notwendig für die moralische Rechtfertigung der Zwangsmaßnahme, d. h.: Wenn eines oder mehrere dieser Kriterien nicht erfüllt sind, ist die Durchführung der Zwangsmaßnahme moralisch nicht gerechtfertigt. Während in Situationen von Fremdgefährdung die Erfüllung der drei genannten Kriterien hinreichend für die Rechtfertigung einer Zwangsmaßnahme ist, müssen in Situationen von Eigengefährdung die folgenden zwei Kriterien zusätzlich erfüllt sein:

- Die von der Zwangsmaßnahme betroffene Person ist im Hinblick auf die medizinische Maßnahme trotz geleisteter Entscheidungsassistenz nicht einwilligungsfähig (Kriterium der Einwilligungsunfähigkeit).
- Die Zwangsmaßnahme erfolgt im Einklang mit dem vorausverfügten oder mutmaßlichen Willen der betroffenen Person (Kriterium des vorausverfügten oder mutmaßlichen Willens).

In Situationen von Eigengefährdung ist die Erfüllung aller fünf Kriterien hinreichend für die moralische Rechtfertigung der Zwangsmaßnahme. Zugleich ist jedes Kriterium für sich genommen notwendig, woraus folgt, dass wenn eines oder mehrere dieser Kriterien nicht erfüllt sind, die Durchführung der Zwangsmaßnahme moralisch nicht gerechtfertigt ist. Den Rechtfertigungstest für Zwangsmaßnahmen in Situationen von Eigengefährdung haben wir ausführlicher in Form des von uns vertretenen „kombinierten Modells der Entscheidungsassistenz“ an anderer Stelle dargestellt [27].

## Fazit für die Praxis

- Die Kenntnis ethischer Grundbegriffe und Begründungsansätze ist wichtig für die Identifikation und Analyse moralischer Probleme in der Psychiatrie und sollte in der Ausbildung von Ethikberater*innen stärker vermittelt werden.
- Moralische Probleme müssen von anderen Problemen abgegrenzt werden, um in der klinischen Praxis angemessene Problemlösestrategien wählen zu können.
- Zur ethischen Entscheidungsfindung in Situationen von Eigen- oder Fremdgefährdung ist der Rekurs auf spezifische ethische Rechtfertigungstests auf Grundlage des schwachen Paternalismus bzw. des Schadensprinzips hilfreich.
